# Prospective superficial EPR in-vivo dosimetry study during hypofractionated radiotherapy of breast cancer patients treated with helical tomotherapy

**DOI:** 10.1186/s13014-021-01938-8

**Published:** 2021-10-30

**Authors:** Sebastian Höfel, Matteo Gandalini, Michael K. Fix, Malte Drescher, Felix Zwicker

**Affiliations:** 1 Department of Chemistry and Konstanz Research School Chemical Biology, University of Konstanz, Konstanz, Germany; 2 Klinik und Praxis für Strahlentherapie am Klinikum Konstanz, Konstanz, Germany; 3 Division of Medical Radiation Physics and Department of Radiation Oncology, Inselspital, Bern University Hospital and University of Bern, Bern, Switzerland; 4 Department of Radiation Oncology, Heidelberg University Hospital, Heidelberg, Germany; 5 Clinical Cooperation Unit Molecular Radiation Oncology, German Cancer Research Center (DKFZ), Heidelberg, Germany; 6 Department of Chemistry, AG Drescher, University of Konstanz, Universitätsstraße 10, Box 706, 78457 Konstanz, Germany

**Keywords:** In vivo, EPR dosimetry, Lithium formate, Alanine, Breast cancer, Radiotherapy, IMRT, Tomotherapy, Hypofractionated

## Abstract

**Background:**

In-vivo dosimetry (IVD) is a patient specific measure of quality control and safety during radiotherapy. With regard to current reporting thresholds for significant occurrences in radiotherapy defined by German regulatory authorities, the present study examines the clinical feasibility of superficial electron paramagnetic resonance (EPR) IVD of cumulative total doses applied to breast cancer patients treated with helical intensity-modulated radiotherapy (tomotherapy).

**Methods:**

In total, 10 female patients with left- or right-sided breast cancer were enrolled in this prospective IVD study. Each patient received a hypofractionated whole breast irradiation. A total median dose of 42.4 Gy in 16 fractions (5 fractions per week) was prescribed to the planning target volume. The treatments were completely delivered using helical tomotherapy and daily image guidance via megavoltage CT (MVCT). For each patient, three EPR dosimeters were prepared and placed at distinct locations on the patient’s skin during the delivery of all fractions. Two dosimeters were placed next to the ipsilateral and contralateral mammilla and one dosimeter was placed ventrally to the thyroid (out-of-primary-beam). The total doses delivered to the dosimeters were readout after all fractions had been administered. The measured total dose values were compared to the planned dose values derived from the treatment planning system (TPS). Daily positional variations (displacement vectors) of the ipsilateral mammilla and of the respective dosimeter were analyzed with respect to the planned positions using the daily registered MVCT image.

**Results:**

Averaged over all patients, the mean absolute dose differences between measured and planned total dose values (± standard deviation (SD)) were: 0.49 ± 0.85 Gy for the ipsilateral dosimeter, 0.17 ± 0.49 Gy for the contralateral dosimeter and -0.12 ± 0.30 Gy for the thyroid dosimeter. The mean lengths of the ipsilateral displacement vectors (± SD) averaged over all patients and fractions were: 10 ± 7 mm for the dosimeter and 8 ± 4 mm for the mammilla.

**Conclusion:**

Superficial EPR IVD is suitable as additional safeguard for dose delivery during helical tomotherapy of breast cancer. Despite positional uncertainties in clinical routine, the observed dose deviations at the ipsilateral breast were on average small compared to national reporting thresholds for total dose deviations (i.e. 10%/4 Gy). EPR IVD may allow for the detection of critical dose errors during whole breast irradiations.

## Introduction

In radiotherapy, tumor control as well as the occurrence of deterministic side effects are, in general, very sensitive to the absorbed dose within the tumor and normal tissue, respectively [1]. Therefore, accuracy requirements of radiation dose delivery to patients are high [1–3].

Each step and component within the applied radiotherapy treatment chain introduce errors and uncertainties that affect the accuracy of the actually delivered dose to the patient [4]. Especially intensity modulated radiotherapy (IMRT) treatments are complex and highly individualized with respect to radiation delivery [5]. IMRT treatment plans are tailored towards optimized organs at risk (OARs) sparing and conformal dose coverage of planning target volumes (PTVs), which often leads to steep dose gradients in the surrounding body regions. The delivered cumulative dose distribution within a patient undergoing fractionated radiotherapy may be different from the planned dose distribution due to anatomical changes during the treatment course (e.g. tissue swelling or shrinkage), intra- and inter-fractional organ motion as well as setup errors of the patient with respect to the treatment beam [6]. Nowadays, image guided radiotherapy (IGRT) is frequently applied to reduce setup errors and by this means to improve the geometric accuracy and uncertainty of dose administration [7]. Moreover, IGRT allows to monitor anatomical changes during the treatment course. However, IGRT is not capable of perfectly reproducing the planned irradiation situation as defined in the treatment plan on each treatment day. Variabilities of the body’s outline, daily positional variations of OARs and target volumes etc. need to be tolerated to some extent.

Since recently, a central reporting system for significant occurrences in radiotherapy has been established in Germany [8, 9]. Severe deviations of the actually delivered cumulative dose from the planned total dose for both, target volumes and OARs may constitute a reportable event. Reporting thresholds are defined in the current radiation protection ordinance [10] and relate to cumulative total doses actually delivered to patients. For target volumes, total dose deviations of ± 10% from the planned mean dose need to be reported. Also local total dose deviations of ± 10% with respect to the planned total dose within target structures are defined as thresholds, if, moreover, the absolute value of the dose difference exceeds 4 Gy. For OARs, reporting thresholds relate to the planned total mean dose and to the total dose constraints defined in the institution’s standard operating procedures. Reporting criteria are met, if actually delivered total radiation doses to OARs exceed these values by more than 10%.

In-vivo dosimetry (IVD) is a valuable method for quality assurance in radiotherapy that complements pretreatment quality checks by determining actually delivered radiation doses. So far, point detectors are usually applied during one or a few fractions at representative locations inside body cavities or at the skin level. By this means, IVD is utilized as an additional and independent check of the delivery of single fraction doses to organs at risk and/or to target volumes [4, 11–13]. Moreover, IVD is used for dose measurements outside the primary beam (out-of-field), where clinical treatment planning system (TPS) show accuracy limitations [14]. Although regulatory requirements in Germany imply IVD of cumulative total doses delivered during the complete radiotherapy treatment course, IVD has not yet been routinely applied for this purpose. An appropriate IVD system must meet high accuracy requirements for the determination of cumulative total doses in radiotherapy in order to reliably detect dose errors within the defined reporting thresholds.

Electron paramagnetic resonance (EPR) dosimetry (also known as electron spin resonance (ESR) dosimetry) provides many beneficial features for measuring cumulative total doses in-vivo [15]. While traditionally L-alanine (ALA) was used for EPR dosimetry in radiotherapy [16–20], recent studies investigated the applicability of novel detector materials such as lithium formate monohydrate (LFM) in the radiotherapy dose range [21–23]. Compared to ALA, LFM offers higher dose precision (< 3%) down to doses of around 1 Gy when applying a practical measuring protocol (10 min readout time per pellet) tailored for routine clinical use in radiotherapy [23]. However, EPR dosimetry and especially EPR IVD is rarely applied in radiotherapy so far.

In a recent proof of principle study, we utilized a rigid anthropomorphic phantom and demonstrated the suitability of superficial EPR IVD using LFM (and ALA) for measuring cumulative total doses during complex head and neck IMRT treatments [24]. Based on the findings of our previous work we concluded that superficial EPR IVD is particularly suitable for total target dose verification, especially when treating near-surface targets. Radiotherapy of the female breast is supposed to be an obvious clinical application example. As a matter of fact, dosimetric treatment verification of whole breast irradiations performed with IMRT is of special interest, since inter- and intra-fractional motion such as breathing [25, 26], organ swelling or shrinkage [27] as well as variabilities regarding positioning of the patient [25] may affect the actually delivered dose.

The primary aim of the current study was to demonstrate the clinical feasibility of superficial EPR IVD for verifying cumulative total doses delivered during real breast IMRT treatments with regard to current reporting thresholds for significant occurrences. The method is exemplarily demonstrated in the high dose range (next to the PTV), in the intermediate OAR dose range (at the contralateral breast) and in the low dose range (out-of-primary-beam). Three feasibility aspects are reported: (i) Practicability of superficial dosimeter positioning in clinical routine, (ii) deviations between measured and planned total dose values when dosimeters are exposed to daily positional variations and (iii) the magnitude of these dose deviations in relation to current reporting thresholds.

For this purpose, superficial EPR IVD was performed during 10 real IMRT breast treatments. All patients received a hypofractionated whole breast IMRT treatment using helical tomotherapy and daily image guidance via megavoltage CT (MVCT). Cumulative total doses were measured superficially by placing EPR dosimeters next to the left and right mammillae as well as ventrally to the thyroid (out-of-primary-beam) during all fractions. Each EPR dosimeter consisted of one ALA and one LFM pellet. The measured total dose values were compared to the planned total dose values derived from the TPS dose calculation. Daily positional variations of the ipsilateral mammilla and the respective EPR dosimeter were evaluated using the daily registered MVCT image. Deviations between measured and planned dose values at the dosimeter locations were assessed in the context of current national reporting thresholds for significant occurrences in radiotherapy.

## Patients and methods

### Patients and treatment prescription

Ten female patients diagnosed with left- or right sided, nodal negative breast cancer were enrolled in this in-vivo dosimetry study. All patients underwent breast-conserving surgery and were indicated for adjuvant whole breast radiotherapy. All treatments were performed according to the institution’s standard operating procedures. Each patient received a hypofractionated whole breast irradiation using helical tomotherapy and daily IGRT via MVCT imaging. A total median dose of 42.4 Gy in 16 fractions (5 fractions per week) was prescribed to the PTV. The main focus of daily image registration was the correct positioning of the ipsilateral chest wall with respect to the radiation beam. The volume size of the PTV as defined on the planning CT image ranged from 1051 to 2623 ccm. Averaged over all patients, the PTV size was 1596 ccm.

### EPR dosimeters and superficial placement

**Fig. 1:**
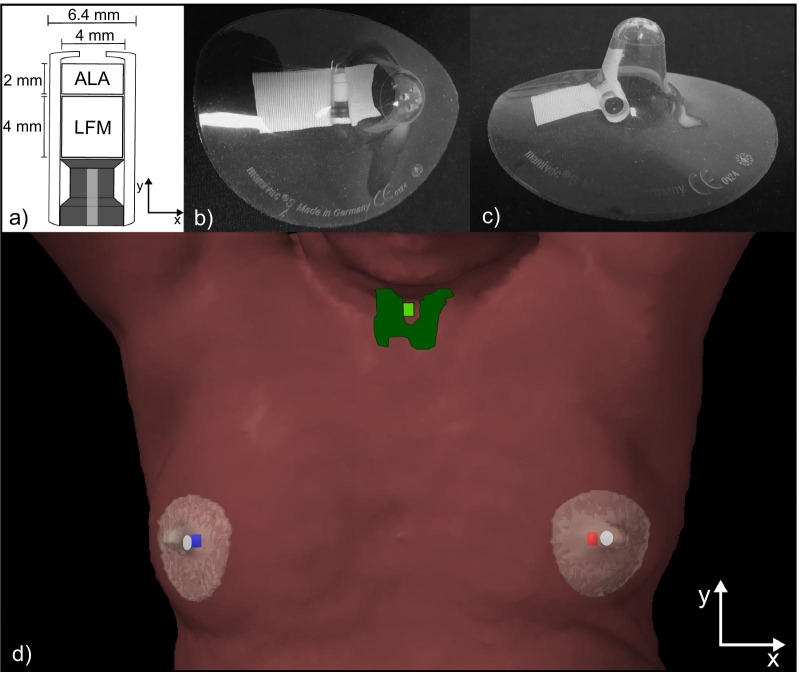
Sketch of cross-section parallel to the EPR dosimeters’ symmetry axis and dimensions of the dosimeter design (**a**). Pictures of an EPR dosimeter affixed inside the cavity of a nipple shield for quick and easy surface application and removal (**b**, **c**). Exemplary 3D view of a left-sided breast cancer patient’s body (brown surface) equipped with three EPR dosimeters (**d**). The ipsilateral EPR dosimeter (iDos) and the contralateral EPR dosimeters (cDos) are shown as red and blue structures, respectively. These dosimeters were placed medial with respect to the left and right mammilla (white structures) using nipple shields (translucent structures). The thyroid and the corresponding EPR dosimeter (tDos) are shown as dark and bright green structures, respectively. Definition of the IEC coordinate system in relation to the dosimeters’ geometry and the patient setup (**a**, **d**)

The EPR dosimeters consisted of a cylindrical polypropylene capsule (outer diameter of 6.4 mm, length of 12 mm) containing one ALA and one LFM pellet (see Fig. 1a). The radiation-sensitive pellets had a cylindrical shape with a diameter of 4 mm and a height of 2 mm (ALA) or 4 mm (LFM). Further details regarding dosimeter design and preparation can be found in previous publications [23, 24].

For each patient, three EPR dosimeters were prepared and placed at distinct locations on the patient’s skin during acquisition of the initial kV-CT used for treatment planning (see “Treatment planning” section) and during the delivery of all MVCTs and all treatment fractions. The EPR dosimeters were attached to clear anatomical points of reference: Two EPR dosimeters (labeled as iDos and cDos) were placed next to the ipsilateral and contralateral mammilla (labeled as iMam and cMam), respectively. Quick and easy dosimeter application and removal was realized by using silicone nipple shields (mamivac®, KaWeCo GmbH, Ditzingen, Germany) enclosing the EPR dosimeters (Fig. 1b, c). The third EPR dosimeter was placed in the vicinity of the thyroid, i.e. just below the laryngeal prominence. The thyroid dosimeter (tDos) was attached to the skin using skin-friendly tape (Micropore™ surgical tape, 3 M Corp., Saint Paul, USA). An example of the dosimeters’ placements is shown in Fig. 1d. Great care was taken that the dosimeters’ symmetry axis was always in parallel with the y-direction of the International Electrotechnical Commission (IEC) accelerator coordinate system displayed in Fig. 1.

Between fractions, the EPR dosimeters were stored in an air-tight box located outside the treatment room. The storage box provided a constant level of 34 ± 2% relative humidity in order to minimize possible EPR signal fading over the course of treatment.

### Treatment planning

Computed tomography (CT) datasets were acquired for treatment planning. Image reconstruction was performed with a 2 mm slice thickness. All patients were positioned in supine position with arms raised above the head. A wedged immobilization system (wingSTEP™ and MCT wedge evo indexed, IV-T, Innsbruck, Austria) was used in order to increase the reproducibility of patient positioning.

The following OARs were contoured and considered during treatment planning: The left and right lungs, the contralateral breast and the heart. The thyroid was contoured as region of interest (ROI) and was located out-of-field. The PTV was defined according to the institution’s standard operating procedures; depending on the diagnosis (left/right sided breast cancer), the PTV encompassed the whole left or right mammary gland and always included the chest wall. A safety margin of 2 cm in cranio-caudal direction was considered during the PTV delineation process. For the purpose of skin sparing, PTV delineation was restricted to a depth of 3 mm under the skin of the ipsilateral breast. The 3 mm strip between the PTV and the breast surface was contoured as well (TV_Surface_) and considered during optimization as separate target volume.

Helical IMRT plans were generated for a Tomotherapy Hi-Art® treatment machine by using Accuray’s integrated TPS Hi-Art® PlanningStation 5.1.1.6 (Accuray Inc., Sunnyvale, CA, USA). All plans were created with the following settings: Longitudinal field width of 2.5 cm (dynamic jaw mode (TomoEdge™)), pitch of 0.282 and planning modulation factors between 2.6 and 2.9. During optimization, plan normalization was set to D_50%_(PTV) = 42.4 Gy corresponding to the prescribed total dose. Final dose calculation was performed with a fine dose grid, i.e. the dose voxel size was 2 mm along all three IEC coordinate axes.

**Fig. 2:**
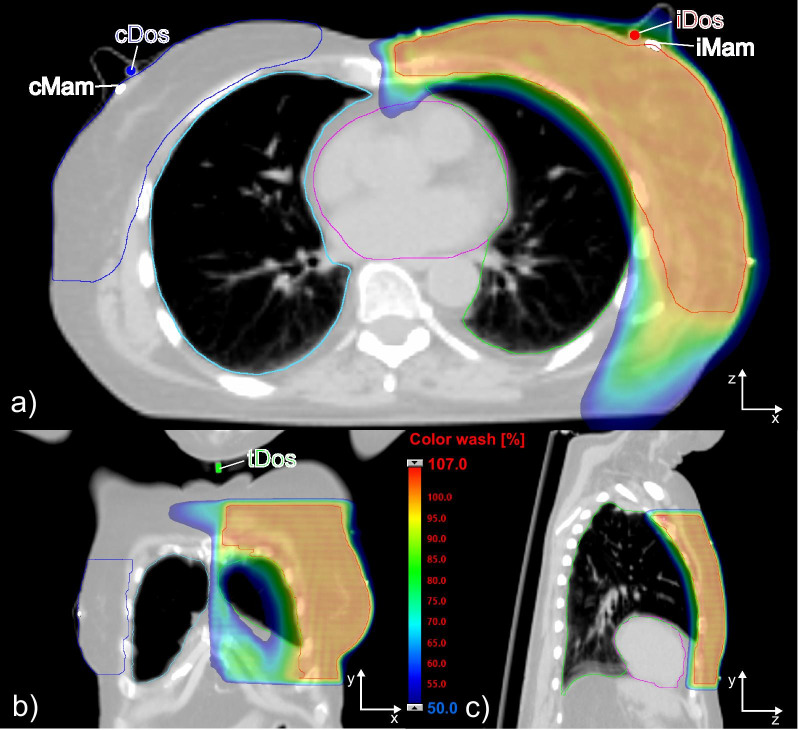
Axial (**a**), coronal (**b**) and sagittal (**c**) views of the calculated dose distribution superimposed on the planning CT dataset for an exemplary left-sided breast cancer patient (Pat#10). The ipsilateral dosimeter (iDos) and contralateral dosimeter (cDos) are shown in (**a**) as red and blue spots, respectively. The right mammilla (cMam) and the left mammilla (iMam) are marked as white segments. The out-of-field location of the thyroid dosimeter (tDos) is illustrated in (**b**). Contours of the PTV (red) and OARs (ipsilateral lung (green), contralateral lung (cyan), contralateral breast (blue) and heart (purple)) are displayed. The dose distribution is shown in colorwash ranging from 50 to 107% of the prescribed dose

During plan optimization, radiation doses to the OARs were minimized focusing primarily on the dose to the ipsilateral lung while covering at least 99% of the PTV with a minimum dose of 95% of the prescribed dose. Dose maxima were restricted to 107% of the prescribed dose. All treatment plans were clinically acceptable. Based on the TPS final dose calculations, Table 1 summarizes the planned mean doses within the target structures and OARs/ROI for all ten patients included in this study. Figure 2 shows the planned dose distribution in three orthogonal planes superimposed on the corresponding CT dataset for an exemplary patient. Figure 2 also illustrates the location of the superficially applied EPR dosimeters in relation to the patient’s anatomy and to the planned dose distribution.

**Table 1:** Mean doses to the targets and OARs/ROI after final dose calculation for all ten patients together with the arithmetic mean doses (± standard deviation (SD)) over all patients

Pat #—right (R)/left (L) side	Mean dose to targets and OARs/ROI [Gy]						
	PTV	TV_Surface_	Lung (ipsilat.)	Lung (contralat.)	Heart	Breast (contralat.)	Thyroid
1—L	42.3	37.6	9.8	3.3	5.9	3.5	1.0
2—L	42.2	37.2	11.0	4.6	6.8	5.2	1.3
3—L	42.2	37.4	10.1	3.9	5.6	4.6	2.9
4—L	42.0	37.1	9.7	3.4	6.1	6.4	1.1
5—R	42.2	37.7	11.0	4.8	5.7	5.1	0.9
6—R	42.2	36.7	10.1	3.9	6.5	5.0	0.9
7—L	42.2	37.8	11.4	4.1	5.5	6.4	2.0
8—L	42.2	37.2	11.1	4.1	7.6	5.4	1.3
9—L	42.2	37.3	11.4	4.6	7.5	4.2	2.6
10—L	42.3	37.3	12.0	4.3	7.6	4.1	1.3
Average (± SD)	42.2 (± 0.1)	37.3 (± 0.3)	10.8 (± 0.8)	4.1 (± 0.5)	6.5 (± 0.8)	5.0 (± 0.9)	1.5 (± 0.7)

### Superficial dosimetry and uncertainty considerations

Planned total dose values *D*^*p*^ and measured total dose values *D*^*m*^ as well as the associated dose uncertainties were obtained according to the procedures reported previously [23, 24]. The main steps are shortly outlined in the following. All uncertainties in this work are determined and expressed in accordance with the *Guide to the Expression of Uncertainty in Measurement* published by the International Organization for Standardization [28]. Unless otherwise stated, all uncertainties are to be seen as type B standard uncertainties (1σ).

Planned total doses *D*^*p*^ to each EPR pellet were derived from the TPS final dose calculations. ALA and LFM pellets were contoured separately with a diameter of 4 mm. HU values inside the pellet contours were taken as received from the planning CT, i.e. no density override was performed. Due to partial volume artefacts resulting from the finite CT slice thickness (2 mm), two adjacent structures (*S*_*i*_^vc#1^, *S*_*i*_^vc#2^) were assigned to each pellet *i* in order to capture the pellet’s true position (see Fig. 1 in [24]). For each pellet, the mean dose values within these two structures (*D*_*m*_*(S*_*i*_^*vc#*1^), *D*_*m*_(*S*_*i*_^*vc#*2^)) were extracted from the dose statistics table of the TPS. Planned dose values *D*_*i*_^*p*^ for each pellet were defined as the mean value of the mean doses calculated within the two associated structures (*D*_*i*_^*p*^ = *mean*{*D*_*m*_(*S*_*i*_^*vc#*1^)*,D*_*m*_(*S*_*i*_^*vc#*2^)}). An additional imaging dose due to the clinical IGRT procedure was estimated from phantom measurements (see “IGRT imaging dose consideration” section) and was included in the final planned dose value. The combined uncertainty of the planned dose *u*_*c*_(*D*^*p*^) consists of two components: The uncertainty of the TPS dose calculation (2.2% for iDos (in-field), 5.4% for cDos and tDos (lying predominantly out of the primary beam)) and an additional contouring uncertainty component specific to each pellet [24].

Measured total dose values *D*^*m*^ were obtained via EPR dosimetry. EPR measurements were performed using a compact benchtop spectrometer (MiniScope MS 5000, Magnettech by Freiberg Instruments GmbH, Freiberg, Germany) and a practical dosimetry protocol tailored for routine use in radiotherapy [23]. The EPR pellets were readout after all fractions were delivered. The measurement lasted about 10 min per pellet. The EPR measurements were corrected for fading and superficial application [24]. Since the EPR dosimeters were irradiated under less controlled conditions (30 °C ± 5 °C (2σ) assumed for superficial in-vivo application) compared to the dosimeters used for calibration (25 °C ± 2.5 °C (2σ)) an additional temperature correction and uncertainty contribution was considered by applying previously reported correction factors [29]. The combined uncertainty for the measured dose values *u*_*c*_(*D*^*m*^) consisted of four components: The uncertainties of correcting for fading, for superficial application, for the irradiation temperature and the dose dependent uncertainty of the EPR measurement [23, 24].

The combined uncertainty *u*_*c*_(*ΔD*) of the dose difference *ΔD* = *D*^*m* ^− *D*^*p*^ is calculated via *u*_*c*_(*ΔD*)^2^ = *u*_*c*_(*D*^*p*^)^2^ + *u*_*c*_(*D*^*m*^)^2^*.*

### IGRT imaging dose consideration

Based on the findings of previous research [30] it is assumed that the dose response of the applied EPR dosimeters at the imaging beam quality (about 3.5 MV) equals the dose response at the calibration beam quality (6 MV).

Due to sensitivity limitations of the applied EPR dosimetery system below doses of 1 Gy [23], MVCT imaging doses per fraction were estimated by placing lithium fluoride (LiF) thermoluminescence dosimeters (TLD-100) on the surface of tomotherapy’s ‘cheese phantom’—a cylindrical Virtual Water™ phantom with a diameter of 30 cm and a length (IEC y-direction) of 18 cm. The phantom was supposed to represent a human torso and was positioned off-axis, i.e. at IEC coordinates (x = − 2 cm, z = − 2 cm) in relation to the gantry’s rotating axis, thus, mimicking a left-sided breast irradiation setup. Six TLDs were attached superficially in the phantom’s upper left and upper right quadrants—three TLDs on each side representing possible detector positions of the patient study.

TLDs were provided and read out externally by PTW (PTW GmbH, Freiburg i. Br., Germany). Each of the six TLD detectors consisted of a cylindrical TLD chip (diameter of 4 mm, height of 1 mm) that was encapsulated in a cylindrical *polymethyl methacrylate *(PMMA) rod with a length of 2.5 cm and a diameter of 5 mm. All TLD chips were exactly located in the center of the PMMA rods. Expressed in polar coordinates (R, φ, Z) with the origin lying in the center of the phantom, with φ = 0° corresponding to the IEC z direction and with a Z axis in parallel with the IEC y axis, the centers of the TLDs were located at: (R = 15.25 cm, φ = (− 60°, − 50°, − 40°, 40°, 50°, 60°), Z = 0 cm).

In the present study, all MVCTs were performed in ‘coarse’ acquisition mode. In order to improve TLD readout uncertainties, ten ‘coarse’ MVCTs were applied sequentially to the phantom setup. The determined imaging doses to the TLDs per single MVCT are shown in Table 2. The average imaging doses of 1.17 cGy and 0.88 cGy per MVCT were included in the planned dose values (“Superficial dosimetry and uncertainty considerations” section) for the cDos and iDos EPR pellets, respectively.

**Table 2:** MVCT imaging doses to TLDs placed in the right (R) and left (L) upper quadrant of the ‘cheese phantom’ for imaging mode ‘coarse’

TLD # - (R)/(L) side	Imaging doses to TLDs per MVCT [cGy]
	Coarse mode
1—R (− 60°)	1.08
2—R (− 50°)	1.20
3—R (− 40°)	1.24
Average—R	1.17
4—L (40°)	1.03
5—L (50°)	0.93
6—L (60°)	0.69
Average—L	0.88

The relative uncertainty of the TLD measurements was stated by PTW as 2.5% (1σ). We estimated the absolute uncertainty of the total imaging dose (16 fractions) to *u*_MVCT_ = 4 mGy (1σ). Compared to other sources of uncertainty ([23, 24] and Table 3) this contribution is marginal and was therefore neglected.

**Table 3:** Measured and planned total doses to ALA and LFM pellets for all patients

Pat #	iDos		cDos		tDos	
	*D*^*m*^ (*u*_*c*_)	*D*^*p*^ (*u*_*c*_)	*D*^*m*^ (*u*_*c*_)	*D*^*p*^ (*u*_*c*_)	*D*^*m*^ (*u*_*c*_)	*D*^*p*^ (*u*_*c*_)
	Total doses to ALA pellets [Gy]					
1	44.79 (0.34)	45.00 (1.02)	3.24 (0.13)	3.02 (0.15)	1.24 (0.13)	1.14 (0.06)
2	44.00 (0.33)	43.58 (0.97)	6.77 (0.14)	5.99 (0.32)	1.55 (0.13)	1.14 (0.06)
3	41.52 (0.32)	40.38 (0.91)	5.08 (0.13)	5.33 (0.27)	1.06 (0.13)	1.45 (0.08)
4	41.91 (0.32)	40.92 (0.90)	7.75 (0.14)	7.23 (0.38)	1.23 (0.13)	1.27 (0.07)
5	40.17 (0.31)	38.47 (0.96)	5.06 (0.13)	4.50 (0.23)	1.33 (0.13)	1.04 (0.06)
6	38.77 (0.30)	38.68 (0.85)	6.69 (0.14)	6.26 (0.33)	1.04 (0.13)	1.20 (0.07)
7	38.40 (0.30)	36.30 (1.00)	6.19 (0.14)	5.76 (0.30)	1.17 (0.13)	1.12 (0.06)
8	38.48 (0.30)	38.70 (0.85)	4.93 (0.13)	4.62 (0.24)	1.26 (0.13)	1.84 (0.10)
9	41.26 (0.32)	40.67 (0.98)	2.73 (0.13)	3.61 (0.18)	1.72 (0.13)	1.47 (0.09)
10	41.39 (0.32)	41.09 (1.05)	3.77 (0.13)	3.51 (0.20)	1.04 (0.13)	1.22 (0.07)
	Total doses to LFM pellets [Gy]					
1	44.45 (0.42)	44.97 (0.99)	3.11 (0.05)	3.01 (0.15)	1.23 (0.04)	1.22 (0.07)
2	43.53 (0.41)	43.63 (0.97)	6.53 (0.07)	5.96 (0.31)	1.50 (0.04)	1.21 (0.07)
3	41.33 (0.39)	40.66 (0.89)	5.10 (0.06)	5.35 (0.28)	1.15 (0.03)	1.51 (0.08)
4	41.61 (0.40)	40.26 (0.94)	7.50 (0.08)	7.17 (0.39)	1.03 (0.03)	1.35 (0.07)
5	39.19 (0.37)	38.26 (0.87)	4.97 (0.06)	4.37 (0.23)	0.95 (0.03)	1.15 (0.09)
6	38.83 (0.37)	38.57 (0.85)	6.72 (0.07)	6.36 (0.36)	0.97 (0.03)	1.27 (0.07)
7	38.75 (0.37)	36.87 (0.81)	6.19 (0.07)	5.76 (0.30)	1.04 (0.03)	1.19 (0.07)
8	38.41 (0.37)	38.80 (0.85)	4.67 (0.06)	4.68 (0.24)	1.29 (0.04)	2.01 (0.13)
9	41.08 (0.39)	41.06 (0.91)	2.56 (0.04)	3.73 (0.19)	1.49 (0.04)	1.58 (0.09)
10	40.87 (0.39)	41.98 (0.93)	3.67 (0.05)	3.54 (0.18)	1.04 (0.03)	1.30 (0.07)

Absolute combined uncertainties (1σ) are given in brackets

### Positional variations

Daily absolute positions (IEC coordinates) of the ipsilateral dosimeter $$\mathop{v}\limits^{\rightharpoonup} \,_{iDos}^{frac\:j}$$ and of the ipsilateral mammilla $$\mathop{v}\limits^{\rightharpoonup} \,_{iMam}^{frac\:j}$$ in relation to the location on the planning CT ($$\mathop{v}\limits^{\rightharpoonup} \,_{iDos}^{Plan} ,\mathop{v}\limits^{\rightharpoonup} \,_{iMam}^{Plan}$$) were recorded by means of the daily registered MVCT image. Daily displacement vectors ($$\overset{\lower0.5em\hbox{$\smash{\scriptscriptstyle\rightharpoonup}$}}{\Delta v} \,_{iDos}^{frac\:j} = \mathop{v}\limits^{\rightharpoonup} \,_{iDos}^{frac\:j} - \mathop{v}\limits^{\rightharpoonup} \,_{iDos}^{Plan}$$, $$\overset{\lower0.5em\hbox{$\smash{\scriptscriptstyle\rightharpoonup}$}}{\Delta v} \,_{iMam}^{frac\:j} = \mathop{v}\limits^{\rightharpoonup} \,_{iMam}^{frac\:j} - \mathop{v}\limits^{\rightharpoonup} \,_{iMam}^{Plan}$$) were determined for all treatment fractions $$j$$.

The contralateral dosimeter (cDos) and contralateral mammilla (cMam) as well as the thyroid and the corresponding dosimeter (tDos) were located outside the MVCT’s field-of-view.

## Results

### Practicability

From a clinical staff’s perspective, daily application of the EPR dosimeters on the patients’ skin was quick, simple and reproducible, due to the positioning aids used (nipple shields, skin-friendly tape; see “EPR dosimeters and superficial placement” section) and due to clear anatomical points of reference (left/right mammilla, laryngeal prominence), respectively. Superficial dosimeter application was well tolerated by the patients.

### Positional variations

**Fig. 3:**
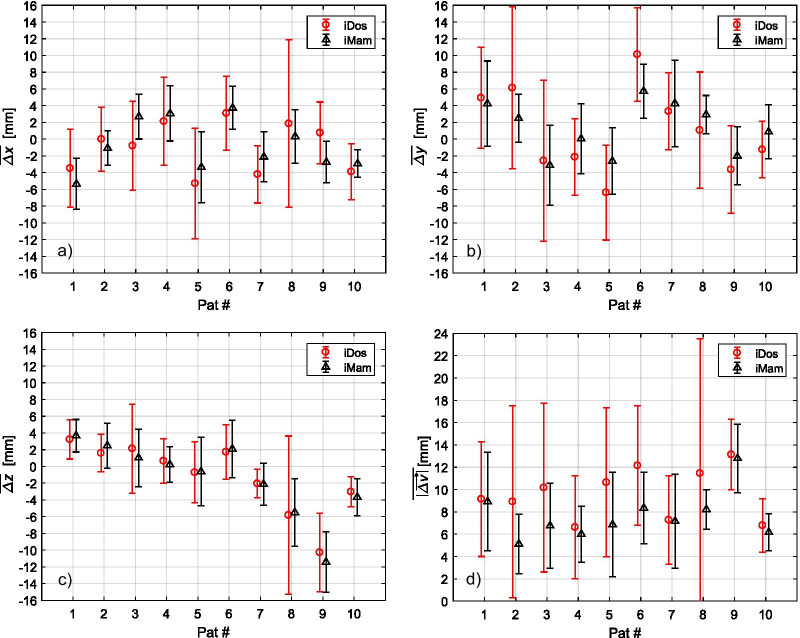
Mean IEC x- (**a**), y- (**b**) and z-component (**c**) of the displacement vectors $$\overset{\lower0.5em\hbox{$\smash{\scriptscriptstyle\rightharpoonup}$}}{{\Delta {\text{v}}}}$$ for iDos (red circles) and for iMam (black triangles) over all fractions and for each patient. Standard deviations (SD) observed over all fractions are indicated by the error bars. Mean lengths of the displacement vector $$\overline{{\left| {\overset{\lower0.5em\hbox{$\smash{\scriptscriptstyle\rightharpoonup}$}}{{\Delta {\text{v}}}} } \right|}}$$ (± SD) over all fraction are shown in (**d**) for each patient

For the ipsilateral mammilla (iMam) and the respective dosimeter (iDos), positional variations with respect to the planned situation were recorded on the basis of the daily registered MVCT images. Figure 3 shows the residual (after applying IGRT patient setup corrections) mean displacement values in IEC x-, y-, and z- direction as well as the mean length of the displacement vector $$\overline{{\left| {\overset{\lower0.5em\hbox{$\smash{\scriptscriptstyle\rightharpoonup}$}}{\Delta v} } \right|}}$$ averaged over all treatment fractions for each patient. Averaged over all patients and fractions, the mean lengths of the ipsilateral displacement vectors (± standard deviation (SD)) were: 10 ± 7 mm for iDos and 8 ± 4 mm for iMam.

The distance (IEC y- component) of the center of the thyroid dosimeter (tDos) to the cranial PTV border was determined on the planning CT image and ranged from 2.0 cm (Pat #8) to 5.0 cm (Pat #5). Averaged over all patients, the mean distance (± SD) was 3.8 ± 0.8 cm.

### Total dose deviations

**Fig. 4:**
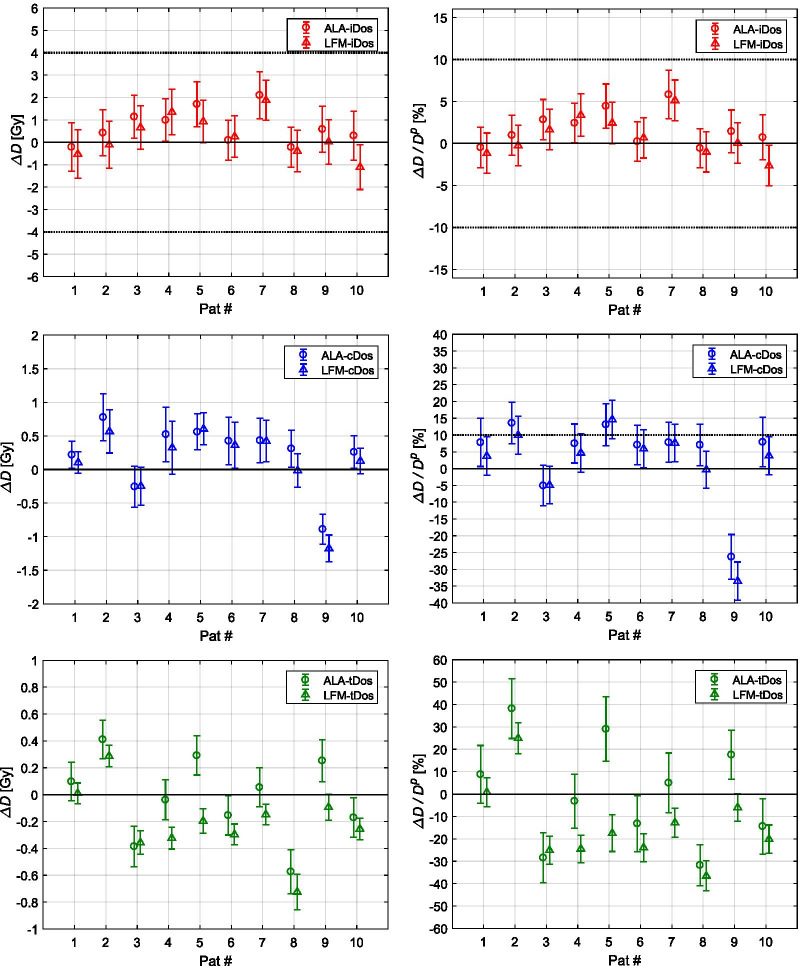
Absolute *ΔD* (left) and relative *ΔD/D*^*p*^ (right) dose differences between measured *D*^*m*^ and planned *D*^*p*^ total dose values for all patients at EPR dosimeter locations: iDos (top), cDos (middle) and tDos (bottom). For comparison, the results obtained via ALA (circles) and via LFM (triangles) are grouped together. Combined uncertainties (1σ) of the dose difference are represented by error bars. Reporting thresholds (10%/4 Gy) are indicated by the black dotted lines where applicable

Measured (*D*^*m*^) and planned (*D*^*p*^) total dose values to the ALA and LFM pellets in combination with absolute combined uncertainties (see “Superficial dosimetry and uncertainty considerations” section) are listed in Table 3 for all three dosimeter locations (iDos, cDos, tDos) and for all patients. Differences between measured and planned dose values observed at each dosimeter location are illustrated in Fig. 4. The dose differences *ΔD* = *D*^*m* ^− *D*^*p*^ are shown in absolute as well as relative terms with respect to the planned dose. Uncertainty margins (1σ) for the dose difference are indicated by the error bars.

The measured total doses to the EPR dosimeters (± SD) averaged over all patients and both pellet types were 40.9 ± 2.1 Gy for iDos, 5.2 ± 1.6 Gy for cDos and 1.2 ± 0.2 Gy for tDos. The observed dose deviations were in the following ranges: From − 1.1 Gy to 2.1 Gy for iDos, from − 1.2 Gy to 0.8 Gy for cDos and from -0.7 Gy to 0.4 Gy for tDos.

Averaged over all patients and both EPR pellet types, the mean absolute dose differences between measured and planned dose values (± SD) were: 0.49 ± 0.85 Gy for the ipsilateral dosimeter, 0.17 ± 0.49 Gy for the contralateral dosimeter and − 0.12 ± 0.30 Gy for the thyroid dosimeter.

### Comparison to reporting thresholds

Dose deviations between measured and planned total dose values were overall small for the ipsilateral dosimeter (iDos) compared to the 10%/4 Gy criterion (Fig. 4).

For the contralateral dosimeter, three out of 20 EPR measurements (Pat#2 and Pat#5) exceeded the planned dose values by more than 10% while the error bars for the dose difference still covered the threshold limit.

## Discussion

In the present prospective IVD study, EPR dosimeters were applied superficially in order to monitor and verify total dose delivery at target, OAR and ROI locations during helical tomotherapy of ten female breast cancer patients, each receiving a complete course of hypofractionated whole breast radiotherapy.

Compared to TLD in-vivo dosimetry [25], EPR dosimetry is suited for measuring total radiation doses accumulated over a complete treatment course. Low signal fading rates, non-destructive readout and highly water equivalent dosimeter materials with negligible dependency on beam quality, dose rate and angle of beam incidence [15] are key features of EPR dosimetry, thus, being suitable for in-vivo application. However, reported EPR IVD studies on patients are rare. EPR IVD using ALA was first reported during total body irradiations [16, 31] and shortly thereafter also in brachytherapy [17, 18]. More recently, ALA dosimetry was performed in body cavities during prostate [19] and gynecological [32] external beam radiotherapy (EBRT). Superficial EPR IVD of OAR doses using ALA dosimeters during volumetric modulated arc therapy (VMAT) of breast cancer patients was shown by Wagner et al. [20]. In their study, the EPR dosimeters were solely applied to the contralateral breast. Thus, doses to the EPR dosimeters were between 3 and 20 Gy, i.e. less than 50% of the prescribed target dose. In the present study, a novel EPR dosimetry material (LFM) was applied during breast EBRT. Besides, ALA pellets were used for comparison purposes. The EPR dosimeters were tested in a wide dose range: close to the PTV receiving high doses in the order of the prescribed dose, on the contralateral breast (intermediate dose) and in front of the thyroid lying out-of-primary-beam (low dose). To our knowledge, in vivo application of LFM dosimeters during patient treatments has not been reported so far. The main advantage of using LFM dosimeters in vivo is their increased sensitivity at lower doses [21–23]. This can be seen from the reduced error bars for the thyroid dosimeter (tDos) in Fig. 4. For tDos, the relative uncertainties (1σ) of the observed dose differences averaged over all patients were 6.6% (LFM) and 12.2% (ALA). Although in this study the EPR dosimeters were read out only once after all fractions were delivered, earlier and repetitive evaluations during the treatment course are basically possible with EPR dosimetry since the dose read-out is non-destructive. By this means, dosimetric verification of target and OAR doses is expected to be feasible also between fractions. In these situations, i.e. at lower doses, LFM dosimeters may show better performance at target and OAR locations compared to ALA.

In the present study, daily image registrations were performed on the ipsilateral chest wall provoking positional variations of the ipsilateral mammilla (iMam) and dosimeter (iDos) with respect to the planned situation (Fig. 3). Despite these positional variations during the course of treatment, total dose differences between measured and planned dose values for the ipsilateral dosimeter (iDos) were below 2.1 Gy (Table 3, Fig. 4). Of course, this claim is subject to the magnitude of positional variabilities. Therefore, positional variations of the ipsilateral dosimeter and of the ipsilateral mammilla were recorded in order to provide a quantitative side condition. Daily positional displacements of the ipsilateral mammilla with respect to the planned position were in the order of 8 ± 4 mm for the IGRT procedure followed in this study (Fig. 3). Similar displacement values were observed for the respective EPR dosimeter (see mean values for iDos and iMam in Fig. 3), however, daily positioning of the dosimeter on the patients’ skin introduced an additional variability leading to increased standard deviations (compare SD values for iDos and iMam in Fig. 3). No significant correlation between the observed dose deviations *ΔD* for iDos and any of the Cartesian components or the length of the recorded mean displacement vector for iDos was found. Pearson correlation coefficients were below 0.3 and corresponding p-values were all above 0.2.

The black dotted horizontal lines in Fig. 4 indicate current reporting thresholds when reporting criteria are translated to the planned total dose values for the EPR dosimeters.

All treatments were performed regularly without special incidents. As a consequence, the results suggest that the observed dose deviations (Fig. 4) are due to daily positional variations and anatomical changes on the one hand and due to dose uncertainties on the other hand.

For the target dosimeter (iDos), the dose deviations and uncertainties were small compared to the reporting thresholds (10%/4 Gy) featuring EPR IVD as a robust technique for critical dose error detection. Overall, total dose deviations detected by EPR dosimetry were mostly within reporting limits. Exceptions occurred for Pat#2 and Pat#5 at the contralateral breast (cDos). Averaged over all patients, however, the relative dose difference *ΔD/D*^*p*^ observed for cDos was 4.0% (ALA) and 1.7% (LFM).

The relative uncertainties (1σ) of the observed dose differences averaged over all patients were 2.4% (LFM) and 2.5% (ALA) for iDos and 5.6% (LFM) and 6.3% (ALA) for cDos, i.e. smaller than the translated reporting threshold of 10% of the planned dosimeter dose.

Absolute total dose deviations for the contralateral dosimeter (cDos) were mostly in the range of ± 1 Gy. Daily positional variations of the contralateral dosimeter and the contralateral mammilla could not be recorded, since the PTV was positioned as close as possible to the machine isocenter during treatment planning and, thus, the daily locations of cDos and cMam were outside the MVCTs field of view. In a previous study it was demonstrated that the applied dose calculation algorithm may underestimate doses to OAR lying predominantly out-of-primary-beam incidence [24]. However, we suppose that the dose deviations observed in this study are rather due to positional uncertainties of the contralateral breast. This assumption is supported by the fact that the negative outliers (Fig. 4, middle) are observed for patients with the largest PTVs (Pat #3: 2623 ccm and Pat #9: 2413 ccm), where positional variations are likely more pronounced. The mean PTV size (± SD) among the remaining eight patients was 1365 ± 260 ccm. We suppose that the observed dose deviations for cDos could have been improved by reducing setup errors of the contralateral breast.

Dose measurements next to the thyroid were performed in order to demonstrate the out-of-field applicability of EPR IVD during radiotherapy treatments. The results show that EPR IVD is capable of measuring low cumulative dose values out-of-primary-beam in the order of 1–2 Gy. However, absolute dose differences *ΔD* between − 0.7 Gy and 0.4 Gy were observed. Relative dose differences *ΔD*/*D*_*p*_ were between ± 40% (Fig. 4) with a relative uncertainty in the order of 6.6% (LFM) and 12.2% (ALA). Daily positional variations of the tDos dosimeter could not be recorded since its position was located outside the daily MVCT scan. It is presumed that the observed dose differences were mainly caused by positional variations (neck setup and tDos positioning). Averaged over all patients, the mean absolute dose differences (± SD) was − 0.12 ± 0.30 Gy, i.e. the scattering of the dose differences is too large and the sample size too small to assess any systematic errors.

The present study demonstrates the clinical feasibility of superficial EPR IVD of total doses delivered during breast cancer treatments. For the ipsilateral dosimeter locations considered in this work, the observed dose differences between measured and planned total doses to the EPR dosimeters were small compared to reporting thresholds. High cumulative total doses (around 40 Gy) at the ipsilateral breast as well as intermediate and lower total doses (between 1 and 6 Gy) at OAR and ROI locations could be measured. A practical EPR dosimetry system tailored for routine clinical use was applied featuring a read out time of 10 min per pellet. The performances of ALA and LFM pellets were comparable in this study. Although not explicitly shown in this study, LFM is expected to be superior for IVD at lower doses (e.g. when measuring single fraction doses at target or OAR locations) due to increased dose precision compared to ALA [23].

## Conclusion

Despite remaining positional uncertainties during image-guided helical tomotherapy of breast cancer, the dose differences between planned and measured cumulative total doses obtained via superficial EPR IVD as well as combined uncertainties of the dose differences were small for the ipsilateral dosimeter compared to current reporting thresholds in radiotherapy. Thus, EPR IVD is suitable and clinically feasible to assist in detecting, preventing and investigating severe dose misadministration to the treated breast according to current reporting criteria. Dose delivery to the contralateral breast and to the thyroid lying out-of-field could be monitored down to approximately 1 Gy cumulative dose. Superficial EPR IVD is to be seen as an additional safeguard for monitoring cumulative total doses to radiotherapy patients. In future clinical routine, superficial EPR IVD could assist in recognizing treatment errors and may support further investigations whether the criteria for reporting are met.

## Data Availability

All original data will be made available upon reasonable request.

## Abbreviations

ALA: L-Alanine
cDos: Contraleteral dosimeter
cMam: Contralateral Mammilla
CT: Computed tomography
EPR: Electron paramagnetic resonance
IEC: International Electrotechnical Commission
iDos: Ipsilateral dosimeter
IGRT: Image guided radiotherapy
iMam: Ipsilateral mammilla
IMRT: Intensity modulated radiotherapy
IVD: In vivo dosimetry
LFM: Lithium formate monohydrate
LiF: Lithium fluoride
MVCT: Megavoltage computed tomography
TLD: Thermoluminescence dosimeter
OAR: Organ at risk
PMMA: Polymethyl methacrylate
PTV: Planning target volume
ROI: Region of interest
SD: Standard deviation
tDos: Thyroid dosimeter
TPS: Treatment planning system
